# Full arch rehabilitation in patients with atrophic upper jaws with zygomatic implants: a systematic review

**DOI:** 10.1186/s40729-021-00297-z

**Published:** 2021-02-26

**Authors:** Ana Helena Pereira Gracher, Marcos Boaventura de Moura, Patrícia da Silva Peres, Geninho Thomé, Luís Eduardo Marques Padovan, Larissa Carvalho Trojan

**Affiliations:** 1 Technical research specialist, Neodent, Curitiba, Brazil; 2 Department of Occlusion, Fixed Prosthodontics and Dental Materials, School of Dentistry, Federal University of Uberlandia, Av. Pará 1720, Bloco 4LB, sala 39, Uberlandia, MG 38405-902 Brazil; 3 Department of Implantology, ILAPEO College, Curitiba, PR Brazil

**Keywords:** Full arch rehabilitation, Implant, Prosthetic rehabilitation, Surgery, Zygomatic implant

## Abstract

**Background:**

The main objective of this systematic review was to present the outcomes of the treatment with zygomatic implants (ZIs) in the rehabilitations of atrophic upper jaw.

**Findings:**

An electronic database search in PubMed, along with a manual search, taking into account language and study period, was performed by two observers; any type of clinical trial and series that included the use of ZIs was used. In the search strategy, the following search terms were used: zygom* AND dental (Implant OR implants) AND edentulous NOT (biomechanic* OR finite element) NOT cadaver. The search was limited to English language, full text, and humans. Literature reviews and clinical case reports were not considered. Forty-two articles published between March 2003 and April 2019 were included in this analysis. The cases of 1247 patients were recovered; these patients received 2919 ZIs. Fifty-two ZIs were removed during the follow-up time. The survival rate of these implants was 98.22%, with a minimum follow-up of 1 month and a maximum of 228 months. Different surgical techniques were used to place ZIs; however, the intrasinusal technique was the most used (23 studies). Post-surgical sinusitis was the most common complication reported in the studies (39 cases).

**Conclusions:**

Based on this review, ZIs were commonly used for rehabilitation of patients with atrophic upper jaw. The survival rates presented were high, and the surgical technique is dependent on the professional experience and the local anatomy. However, it needed additional clinical evidence on bone resorption, esthetic outcomes, and physiological characteristics.

## Introduction

The loss of posterior maxillary alveolar bone results in reduction of the residual ridge. This zone usually exhibits poor bone quality, resulting in lack of primary stability and may compromise osseointegration [1]. The presence of inadequate bone quantity has implicated in several procedures of bone augmentation, such as maxillary sinus elevation and bone block graft; both of which may involve several surgical procedures. On the other hand, the technique of zygomatic implants (ZIs) results in less invasive and more predictable procedures [1–3].

Several techniques have been proposed to resolve the maxillary atrophy, as elevation of the maxillary sinus floor, surgical maxillary reconstruction with iliac crest, cortical plate expansion, osteotome sinus lifting, bone grafts, titanium meshes, or Le Fort I maxillary down fracture [1, 4, 5]. Some of these treatment options need multiple surgical interventions, varying success rates and increased surgical fees [6]. The treatment with fixtures of patients with severe atrophy is more hazardous and sometimes, impossible without bone grafting [7]. A retrognathic maxilla may require a Le Fort I osteotomy and bone grafting in order to increase bone volume for implants and correct facial morphology, while onlay and/or inlay bone grafting may be sufficient in cases of a normal intermaxillary relation [8].

Brånemark, in 1989, initially developed the ZIs for the rehabilitation of atrophied maxillae in patients with tumors who had undergone total or partial maxillectomy [9]. Currently, ZIs are indicated for dental rehabilitation of atrophic upper jaws. An implant with the following characteristics was initially designed: 45° head, 4.5-mm diameter at its widest part, and a length of 30 to 50 mm. The implant follows an insertion path of the palatal aspect in the alveolar process, following the zygomatic alveolar crest until its anchorage in the malar body [10]. And the amount of bone in the zygomatic arch and in the residual alveolar crest has to be evaluated by computed tomography [2].

Initially, it was recommended to combine two ZIs with conventional implants (CIs), preferably in a semicircular construction and to avoid the use the ZI for only unilateral rehabilitation in the upper jaw [9]. With the development of the techniques, ZIs can be used in patients with totally or partially edentulous maxillary who have insufficient bone volume for placement of CIs posterior to the canine [2]. One to three ZIs can be inserted through the posterior alveolar crest passing through the maxillary sinus, or externally to it, to engage the body of the zygomatic bone in each side of the upper jaw [11].

The main objective of this systematic review was to present the result of the treatment with ZIs in the rehabilitations of atrophic upper jaw. The contributing parameters included in this analysis were the survival rate of the ZIs, the surgical techniques used, and the main complications.

## Materials and methods

The current systematic review was reported following the Preferred Reporting Items for Systematic Reviews and Meta-analysis (PRISMA) statement [12]. The review protocol was registered in PROSPERO (International Prospective Register of Systematic Reviews) hosted by the UK’s National Institute for Health Research (NHS), University of York, Centre for Reviews and Dissemination, under the code CRD42020144836.

### Research question

The clinical question in “PICO” format (*P =* patient problem/population, *I =* intervention, *C =* comparison, *O =* outcomes) in our study was as follows: In patients with atrophic upper jaw, does the placement of ZIs by different techniques compared to CIs present acceptable survival rates?

### Inclusion and exclusion criteria

Inclusion criteria of this systematic review included the following:

- Studies aimed at investigating patients with atrophic upper jaws rehabilitated with ZIs;
- Clinical studies in humans, including prospective, retrospective, and case series studies;
- At least one of the following reported results: clinical, radiographic, and patient-centered;
- Full text available in pdf format;
- Reported in the English language.

The exclusion criteria were as follows:

- Articles published in another language other than English;
- Experimental laboratory studies;
- Animal studies;
- Studies that the main theme was not the rehabilitation of atrophic upper jaws with ZIs;
- Systematic reviews;
- Full text articles were not available on the database search;
- Case reports;
- Duplicate articles;
- Letters to editor;
- Commentaries.

The systematic reviews and reviews of the literature on this topic were excluded because they presented repeated data from prospective and retrospective articles included in the current review.

### Search strategy and study identification

An electronic search was conducted in December 2019 in the PubMed database (National Library of Medicine, National Institute of Health) to collect relevant information on the rehabilitation of atrophic upper jaws with ZIs. We used the computer network of the company Neodent (Brazil) to perform the electronic search of data. In the search strategy, the following search terms were used: zygom* AND dental (Implant OR implants) AND edentulous NOT (biomechanic* OR finite element) NOT cadaver. The search was limited to English language, full text, and humans. Two observers examined the resulting articles in order to discern which complied with inclusion criteria, based on their title and abstract. In the event that both observers did not agree upon evaluation, a third observer undertook the final assessment. The investigators then read the selected full-text articles independently, compared their selections, and resolved any conflicts in selection with a third party.

### Outcome measure

The outcome measure reported in this review was the survival of the ZIs and the surgical techniques used, as reported in each study. Survival of the implant refers to the presence of an implant with or without complications. The failure was defined when the implant was removed. The survival of the implants was calculated from the absolute number of implants placed and lost.

### Data extraction

Tables and figures were used to organize the clinical evidence reported in this review. Data recorded included the author, type of study, follow-up, number of patients, number of implants placed in the upper jaw, surgical technique used, number of implants lost, and number of complications.

## Results

### Study selection

**Fig. 1:**
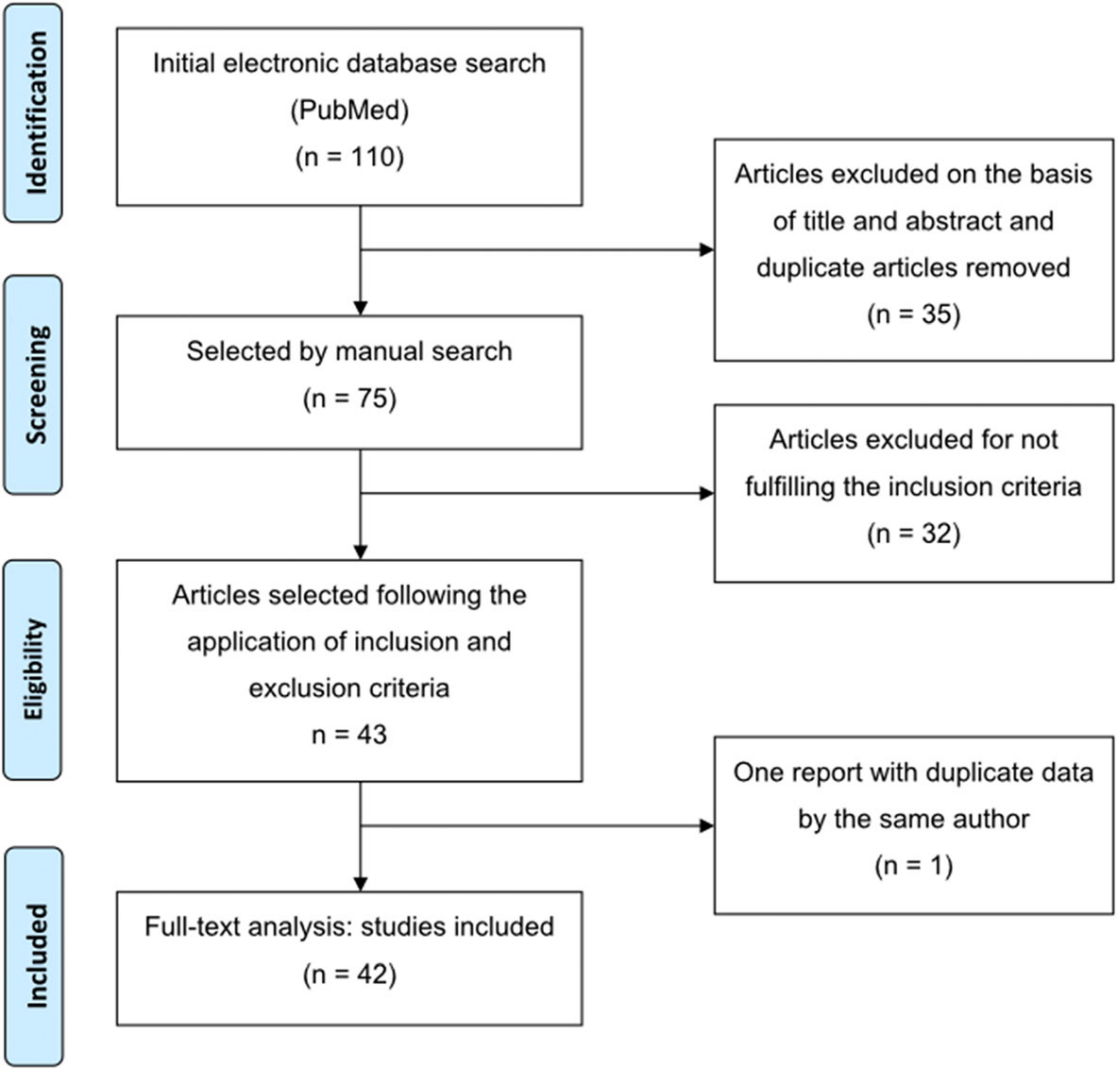
PRISMA flowchart in this systematic review

A hundred and ten articles were identified through electronic searches (PubMed). After analyzing the titles and abstracts and identifying duplicate publications, 35 articles were excluded, leaving 75 for further review. The inclusion and exclusion criteria were then applied, and 43 articles were considered acceptable for full-text analysis. One article with duplicate data from the same author was excluded; thus, 42 articles suitable for inclusion were accepted for systematic review (Fig. 1). The data are presented in Tables 1 and 2: author; type of study; follow-up period (months); number of patients; number of implants placed; surgical technique used; number of non-osseointegrated implants; and number of paresthesias, sinusitis, local infection, and fistulae at implant level were extracted from the 42 selected studies.

**Table 1:** Summary of the studies meeting the eligibility criteria and levels of clinical evidence (CEBM 2011) (*n* = 42)

Author	Type of study	Follow-up (months)	No. of patients	No. of implants	Technique	Levels of evidence
Aleksandrowicz et al. [13]	Retrospective	152	45	88	Intrasinusal and extrasinusal	2a
Alzoubi et al. [14]	Retrospective	228	23	53	NR	2a
Agliardi et al. [4]	Prospective	97	15	42	Intrasinusal	1b
Aparicio et al. [3]	Prospective	72	69	131	NR	1b
Aparicio et al. [8]	Longitudinal cohort study	48	20	36	Extrasinusal	1b
Aparicio et al. [15]	Prospective	72	25	47	Intrasinusal and extrasinusal	1b
Aparicio et al. [16]	Prospective	120	80	197	Intrasinusal and extrasinusal	1b
Aparicio et al. [17]	Prospective	120	22	41	Intrasinusal	2b
Atalay et al. [18]	Prospective	96	16	32	Intrasinusal and extrasinusal	2b
Balshi et al. [19]	Retrospective	NR	77	173	NR	2b
Bedrossian [20]	Case series	6	4	7	Intrasinusal	4
Binon [21]	Case series	108	4	11	NR	4
Brånemark et al. [7]	Prospective	120	28	52	Intrasinusal	1b
Butura and Galindo [22]	Retrospective	24	19	40	Sinus Slot	4
Chana et al. [23]	Retrospective	216	45	88	NR	2a
Chow et al. [24]	Prospective	10	5	10	Intrasinusal	4
Coppedê et al. [25]	Prospective	36	42	94	Extrasinusal	1b
Davó and Pons [26]	Prospective	72	17	68	NR	1b
Davo et al. [27]	Retrospective	29	18	36	Intrasinusal	2b
Davó et al. [28]	Retrospective	12	42	81	Intrasinusal and Sinus Slot	2b
Davó et al. [29]	Retrospective	42	36	71	Intrasinusal and Sinus Slot	2b
Davó [30]	Retrospective	72	24	45	Intrasinusal	2b
Davo et al. [31]	Prospective	12	17	68	Intrasinusal	1b
de Araújo Nobre et al. [32]	Prospective	12	40	72	Extrasinusal	1b
Duarte et al. [6]	Prospective	30	12	48	Intrasinusal	1b
Esposito et al. [33]	Prospective	12	20	80	Intrasinusal	1b
Fernández et al. [34]	Retrospective	48	80	244	Intrasinusal	2b
Fortin [35]	Retrospective	156	49	107	NR	2a
Kahnberg et al. [36]	Prospective	36	76	145	Intrasinusal	1b
Malevez et al. [37]	Retrospective	48	55	103	Extrasinusal	2b
Maló et al. [38]	Prospective	18	29	67	Extrasinusal	1b
Maló et al. [39]	Retrospective	72	39	92	Extrasinusal	2b
Mozzati et al. [40]	Prospective	30	10	40	Intrasinusal	1b
Neugarten et al. [41]	Retrospective	60	28	105	Intrasinusal and extrasinusal	2b
Nocini et al. [42]	Prospective	20	4	16	NR	4
Pellicer-Chover et al. [43]	Retrospective	144	22	44	Sinus Slot	2b
Peñarrocha et al. [44]	Retrospective	84	NR	4	Sinus Slot	2b
Schiroli et al. [45]	Prospective	39	4	7	Intrasinusal	4
Stiévenart and Malevez [46]	Retrospective	40	20	80	Intrasinusal	2b
Wang et al. [47]	Retrospective	NR	15	52	Intrasinusal	2b
Wu et al. [48]	Prospective	36	10	20	NR	1b
Yates et al. [49]	Retrospective	120	25	43	Modified sinus Slot	2a

*NR* not reported

**Table 2:** Summary of studies presenting about non-osseointegration and surgical complications related to the use of zygomatic implants (*n* = 42)

Author	Non-osseointegrated implants	Paresthesia	Sinusitis	Local infection	Fistulae at implant level
Aleksandrowicz et al. [13]	1	NR	4	3	NR
Alzoubi et al. [14]	0	NR	2	0	0
Agliardi et al. [4]	0	NR	NR	NR	NR
Aparicio et al. [3]	0	6	3	0	NR
Aparicio et al. [8]	0	NR	0	0	0
Aparicio et al. [15]	0	NR	NR	0	0
Aparicio et al. [16]	7	NR	NR	6	NR
Aparicio et al. [17]	0	NR	NR	2	NR
Atalay et al. [18]	2	NR	NR	NR	NR
Balshi et al. [19]	6	NR	NR	NR	NR
Bedrossian [20]	0	NR	NR	NR	NR
Binon [21]	0	NR	NR	NR	NR
Brånemark et al. [7]	2	NR	0	1	0
Butura and Galindo [22]	0	NR	0	NR	NR
Chana et al. [23]	5	NR	0	NR	NR
Chow et al. [24]	0	NR	NR	NR	NR
Coppedê et al. [25]	1	NR	NR	NR	NR
Davó and Pons [26]	3	NR	2	NR	1
Davo et al. [27]	0	NR	NR	NR	NR
Davó et al. [28]	0	NR	1	NR	1
Davó et al. [29]	0	NR	0	NR	0
Davó [30]	1	NR	5	NR	NR
Davo et al. [31]	1	NR	0	1	NR
de Araújo Nobre et al. [32]	2	NR	NR	1	NR
Duarte et al. [6]	1	NR	NR	NR	NR
Esposito et al. [33]	2	NR	NR	2	NR
Fernández et al. [34]	1	1	6	NR	1
Fortin [35]	0	NR	0	NR	NR
Kahnberg et al. [36]	5	1	1	NR	3
Malevez et al. [37]	0	NR	1	NR	NR
Maló et al. [38]	1	NR	4	NR	NR
Maló et al. [39]	0	NR	5	NR	1
Mozzati et al. [40]	0	0	0	0	0
Neugarten et al. [41]	4	NR	NR	NR	NR
Nocini et al. [42]	0	NR	0	NR	NR
Pellicer-Chover et al. [43]	1	NR	NR	NR	NR
Peñarrocha et al. [44]	0	NR	NR	NR	NR
Schiroli et al. [45]	0	NR	NR	NR	NR
Stiévenart and Malevez [46]	3	1	1	3	NR
Wang et al. [47]	0	NR	0	NR	NR
Wu et al. [48]	0	NR	NR	NR	NR
Yates et al. [49]	4	NR	1	NR	NR

*NR* not reported

### Study characteristics and quality assessment

Twenty were prospective studies [3, 4, 6, 7, 15–18, 24–26, 31–33, 36, 38, 40, 42, 45, 48], nineteen were retrospective studies [13, 14, 19, 22, 23, 27–30, 34, 35, 37, 39, 41, 43, 44, 46, 47, 49], two were case series [20, 21], and one was a longitudinal cohort study [8]. The articles were classified according to the levels of evidence (based on the University of Oxford’s Center for Evidence Based Medicine criteria) (Table 1) [50].

Different types of studies were included in this review. Overall, this systematic review analyzed 1247 patients with 2919 ZIs placed and 52 loss implants. The survival rate of these ZIs was 98.22%, with a minimum follow-up of 1 month and a maximum of 228 months. The osseointegration period, the surgical technique, and the follow-up period varied between the same types of study and between the different reports. Criteria for treatment success also varied. Of the 42 articles reviewed, there was no randomized controlled clinical trial. One of the 42 articles did not make clear how many patients received ZIs [44], while all articles reported the number of ZIs at each stage of the study. Twelve articles reported the number of failed ZIs and the reasons given (Tables 1 and 2) [6, 7, 13, 16, 26, 30, 31, 33, 34, 36, 38, 46].

The surgical technique most used in this review was the intrasinusal, reported in twenty-three studies [4, 6, 7, 13, 15–18, 20, 24, 27–31, 33, 34, 36, 40, 41, 45–47]. In eleven studies, the extrasinusal technique was used [8, 13, 15, 16, 18, 25, 32, 37–39, 41], five used a combination of intrasinusal and extrasinusal [13, 15, 16, 18, 41], two used intrasinusal and sinus slot [28, 29], five used sinus slot [22, 28, 29, 43, 44], one used sinus modified slot [49], and nine studies did not report which technique was used to implant the implants [3, 14, 19, 21, 23, 26, 35, 42, 48].

Postoperative sinusitis was the most common complication reported in the studies, occurring in 39 cases [3, 13, 14, 26, 28, 30, 34, 36–39]. Eight cases of local infection were reported [7, 13, 16, 17, 31–33, 46], nine cases of paresthesias [3, 34, 36, 46], and seven fistulae at implant level [26, 28, 34, 36, 39].

## Discussion

This systematic review aimed to evaluate the clinical results of the therapy with ZIs in patients with atrophic upper jaw. The 42 articles included in this review provided reliable evidence for the rehabilitation of patients’ oral function. Despite the comprehensive nature of this review, there was heterogeneity in the literature reviewed. Included studies varied in terms of study design, follow-up time, surgical technique, and outcome assessment method (Table 1).

Different types of studies were included in this review. Twenty were prospective studies [3, 4, 6, 7, 15–18, 24–26, 31–33, 36, 38, 40, 42, 45, 48] nineteen were retrospective studies [13, 14, 19, 22, 23, 27–30, 34, 35, 37, 39, 41, 43, 44, 46, 47, 49], two were case series [20, 21], and one was a longitudinal cohort study [8].

The follow-up time of patients who were rehabilitated with ZIs ranged from 1 (minimum) to 228 months (maximum). Only two studies did not report follow-up time (Table 1) [19, 47].

Treatment with the placement of regular size and length implants in patients with severe atrophy is more dangerous and sometimes impossible without bone grafting [7, 36]. Initially, it was recommended to combine two ZIs with CIs, preferably in a semicircular construction and to avoid the use of the ZIs for only unilateral rehabilitation in the upper jaw [36]. With the development of the techniques, ZIs can be used in patients with totally or partially edentulous maxillary who have insufficient bone volume for placement of CIs posterior to the canine [2].

The Brånemark technique for ZI placement that uses an intrasinus path for implant body is called the classic intrasinusal technique [16, 32, 39]. The implant, a endosseal-threaded implant ranging in length from 30 to 52.5 mm, is placed in stable cortical maxillary buttress bone. The implant has a built-in 45°-angled platform lending to ideal ridge positioning. The placement of 2 ZIs and 4 anterior maxillary implants provides retention and support for a fixed prosthesis with 1 in-office surgical procedure, no bone grafting, no hospitalization, and with predictable success [5, 51]. This technique uses a sinus window for placement of the ZIs [51]. Several evolutions of this surgical technique have aimed to improve control of implant positioning, to improve the bone-implant interface, as well as to reduce soft tissue dissection, postoperative pain, and edema while also trying to obtain a prosthetic improvement regarding the emergence profile [40]. In this current review, the intrasinusal technique was the most reported; 23 studies reported using this technique (Table 1) [4, 6, 7, 13, 15–18, 20, 24, 27–31, 33, 34, 36, 40, 41, 45–47]. In this review, the ZIs placed by this technique showed a survival rate of 98.3% (945 ZIs placed and 16 lost ZIs).

Stella and Warner [52] have described a simplified technique for placement of ZIs in which the antrostomy and lifting of the sinus membrane were not necessary. This technique uses a lateral slot outside the wall of the maxillary sinus, avoiding or minimizing the contact of the implant with the sinus membrane. This technique reduced considerably the surgical time and improved the emergence prosthetic profile, due to moving the implant platform nearer to the bone crest, in an optimal 3-dimensional position for the implant-supported restoration [25]. This procedure, called “Sinus Slot Technique” places the zygoma platform directly over the alveolar ridge, very similar to standard dental implants [51]. In this protocol, a guide window is made through the buttress wall of the maxilla, whereby the zygoma implant is guided through the maxilla to the apex insertion at the junction of the lateral orbital rim and the zygomatic arch [39, 51]. The sinus slot technique was reported in four studies, in 2 of them combined with the intrasinusal technique [28, 29] and in two separate studies [43, 44]. Only one implant was lost from 44 implants placed when the technique was used separately (Table 1) [43, 44]. One study reported the use of a “Modified Sinus Slot Technique,” and 43 ZIs were placed between 2000 and 2006 in 25 patients. Four ZIs were lost during the first year of follow-up [49].

In the extrasinusal approach, no initial window or slot is opened at the lateral wall of the maxillary sinus. The extrasinusal technique allows placement of the implant head at or near the top of the residual crest, which results in a more normal extension of the bridge framework [9, 16]. According to this technique, the ZI is placed outside the maxillary sinus, reducing surgical time and the risk of sinus adverse events, and improving surgical visualization. The prosthetic profile of the restoration is considerably improved, as the emergence of the platform of the ZI is positioned on the crest [25]. Six studies reported the use of the extrasinusal technique to place a total of 464 ZIs in 225 patients [8, 25, 32, 37–39]. Only 4 implants were lost during follow-up, with a survival rate of 99.1% [25, 32, 38]. Three other articles used the extrasinusal technique combined with the intrasinusal technique (Table 1) [15, 16, 18]. The authors reported the placement of 276 ZIs in 121 patients. The survival rate of the implants with the two combined techniques was 96.7% (9 ZIs losses) (Table 2) [15, 16, 18].

An extrasinusal technique uses ZIs with a different design to be placed externally to the maxillary sinus, anchored in the zygomatic bone only with immediate function and covered by soft tissue after emerging profile [32, 39]. This surgical technique further evolved into two additional treatment variations: one with the insertion of 4 extrasinusal ZIs (all-on-4 double zygoma) and other using a combination of 1 to 3 extrasinusal ZIs together with 1 to 3 CIs in a hybrid of the all-on-4 treatment concept [32]. Nowadays, the ZIs can also be used unilaterally in cases where there is sufficient bone for CI placement on one side of the arch and a serious deficiency on the other [39].

The use of 2 ZIs associated with 2 CIs is reported in some studies [24, 39]. Chow et al. treated five patients with ZIs. The ZI was placed through the palatal entry point transmucosally. In addition, 2 CIs were placed in each patient. They were followed by 10 months, and during this time, there was no implant failure [24]. Binon et al. described different sets of successfully treated using ZIs. In the first case, a 60-year-old woman presented with a primary complaint that her maxillary implants were painful. Intake radiographs verified significant cratering and bone loss around all implants. Two CIs and 2 ZIs were placed. In the second case, a 45-year old female patient in need of restoring the upper arch following ablative surgery of a mucoepidermoid tumor was submitted a treatment with 2 anterior CIs and 2 ZIs. A postoperative radiograph taken after more than 5 years of function shows excellent osseous stability. The third case was a male patient with a history of right side cleft palate and lip surgically repaired. The patient was submitted to a zygoma-assisted fixed hybrid prosthesis. The temporary prosthesis was secured and functional following surgery. After 3 months, the left anterior conventional implant failed. A second ZI was then inserted, and the temporary prosthesis was modified to accept an additional temporary coping. Six months from insertion of the second ZI, the implants were tested, and a final impression was obtained. The prosthesis has successfully functioned for more than 9 years [21].

The use of variable number of ZIs associated with a variable number of CIs is found in several reports. Malevez et al. reported a retrospective study of 103 ZIs placed in 55 patients. ZIs were placed in 41 women and 14 men patients. Patients had a combination of 1 or 2 ZIs with 2, 3, 4, 5, or 6 CIs. In patients where 6 implants were placed, 2 were placed in the maxillary tuberosity. None of the 103 ZIs failed at the time of the prosthesis insertion (6 months). Of all the 194 implants placed in the upper jaw, 16 were lost [37]. Brånemark et al. reported that twenty-eight patients were treated. In total, 52 ZIs (range 30–50-mm long) and 106 CIs (range 10–20-mm long) were placed. In 24 of the patients, ZIs were placed bilaterally and in 4 unilaterally. In general, was placed 2–4 implants in the anterior region to obtain adequate mechanical stability for the prosthesis. The ZI was placed in the incisura above zygomatic arch. During the whole follow-up period, 3 implants failed. Of the 106 CIs placed during the insertion of the zygoma fixture, 29 were lost in 13 patients. The overall prosthetic rehabilitation rate was 96% after at least 5 years of function [7]. Several other authors have reported using the variable number of conventional implants associated with ZIs [3, 4, 8, 15, 18, 19, 25, 28–30, 32, 36, 38, 39, 43, 45, 49].

The placement of ZIs without CIs was used in atrophic upper jaws, including 1 ZI, 3 ZIs, or 4 ZIs. Duarte et al. described a surgical/prosthetic protocol for the treatment of extremely atrophic maxillae using 4 ZIs in an immediate loading system. In this study, 48 ZIs were placed in 12 patients. Implants were placed in the body of the zygomatic bone and in the ridge at the rim of the maxilla. At 6-month evaluation, 1 implant was considered unsuccessful, as the removal of the prosthetic abutment was not possible since the implant presented rotational mobility. At the 30-month follow-up, 1 further implant presented rotational mobility. All patients demonstrated great satisfaction with improvements of masticatory, esthetic, phonetic, and psychological conditions [6]. Stiévenart et al. studied 20 patients with extremely resorbed upper jaw provided with 4 ZIs. Eighteen patients followed the same surgical protocol. The anterior implant was first placed, and the emergence was at the level of the second incisor or canine. The posterior ZI and the emergence point of the second implant was at the level of the second premolar–first molar. All the ZIs were stable at the time of the placement. The survival rate of the implants was 96% (77 ZIs of 80), and the 3 failed implants were from the same patient. Only 1 patient had a unilateral sinusitis, which was successfully treated with antibiotics [46]. The placement of ZIs without CIs has been reported in other studies [21, 26, 31, 33, 40, 42].

The overall survival rate of all studies reported in this review was 98.22%. A total of 52 ZI losses occurred during the follow-up time of a minimum 1 month and a maximum of 228 months (Table 1). Some complications were also reported in the studies: 9 cases of paresthesias [3, 29, 35, 38, 47], 39 sinusitis [3, 13, 14, 26, 28, 30, 34, 36–39, 46, 49], 16 local infection [7, 13, 16, 17, 31–33, 46], and 7 fistulae at implant level [26, 28, 34, 36, 39] (Table 2).

The main limitation of our study is the lack of a randomized clinical trial on the subject, which limits the level of evidence of the information obtained. Meta-analysis was not possible due to the heterogeneity of the studies and their reported data.

## Conclusions

In conclusion, ZIs are commonly used for the rehabilitation of patients with atrophic upper jaws. The total of 2919 ZIs were placed in 1247 patients. Only 52 implants were removed during follow-up ranging from 1 to 228 months in the studies. Survival rates of implants are high (98.22% survival after follow-up, reported in this review). Different surgical techniques were used and presented high survival rates of ZIs, varying the use of the professional experience and local anatomy. Some complications may occur in the trans-operative or postoperative period, and the most common is sinusitis.

## Data Availability

The datasets generated and analyzed during the current study are available from the corresponding author upon reasonable request.

## Abbreviations

ZI: Zygomatic implant
CI: Conventional implant
NR: Not reported
PRISMA: Preferred reporting items for systematic reviews and meta-analyses.
